# Effect of Metal Cations on Selenite Bioreduction by a Consortium of Halophilic/Halotolerant Bacteria and/or Yeast

**DOI:** 10.1111/1758-2229.70320

**Published:** 2026-05-19

**Authors:** Elham Lashani, Hamid Moghimi, Raymond J. Turner, Mohammad Ali Amoozegar

**Affiliations:** ^1^ Extremophiles Laboratory, Department of Microbiology School of Biology, College of Science, University of Tehran Tehran Iran; ^2^ Department of Microbiology School of Biology, College of Science, University of Tehran Tehran Iran; ^3^ Microbial Biochemistry Laboratory, Department of Biological Sciences University of Calgary Calgary Alberta Canada

**Keywords:** bioreduction, halophilic/halotolerant microorganisms, metal cations, selenite

## Abstract

Selenium oxyanion pollution in the environment, often originating from anthropogenic industrial activities, causes significant risks to human health and the ecosystem. The selenite bioreduction to the less toxic form of selenium, Se (0), by microbial species and consortia offers a promising approach for bioremediation of polluted environments. This study explores the effects of potentially toxic co‐contaminating metal cations, including cadmium (Cd), lead (Pb), and zinc (Zn), on the bioreduction of selenite by a consortium of halotolerant and halophilic bacteria and yeast. The effectiveness of the consortium in reducing selenite was investigated in conditions that mimic multi‐metal pollution found in the real world, with varying levels of the different metals. The results showed that Pb and Cd had negative effects on the mixed consortium efficacy, while Zn showed an augmenting effect on the bacterial consortia. Also, molybdate significantly reduced selenite removal in all consortia. Kinetic analyses using the Michaelis–Menten model were applied for evaluating the rate of selenite reduction. Also, selenium biosorption and dispersion in microbial cells were studied using electron microscopy and EDS analysis. The findings provide insights into the use of microbial consortia in contaminated environments and highlight the potential for bioremediation strategies that can cope with multiple pollutants.

## Graphical abstract

Evaluation the effects of potentially toxic co‐contaminating metal cations, including cadmium (Cd), lead (Pb), and Zinc (Zn), on the bioreduction of selenite by a consortium of halotolerant and halophilic bacteria and/or yeast.

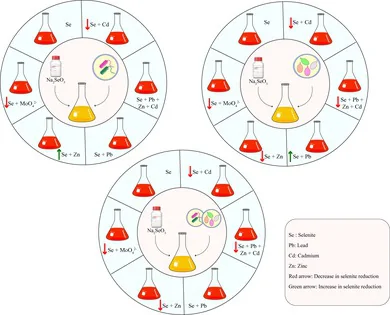

## 1 Introduction

Chemically, selenium is found in coal and sulfur deposits in the geosphere (Coleman et al. 1993). Globally, coal contains between 1.0 and 1.6 mg kg^−1^ of selenium; nevertheless, high amounts of up to 43 mg kg^−1^ have been reported in the US, Russia, Iran, and China (Malvandi 2017; Gerson et al. 2022). Minerals are the main source of selenium in terrestrial systems, although it also comes from anthropogenic activities because of its applications in the different industries, including pharmaceutical and ceramic industries, mining, oil refining, burning coal, and agriculture irrigation. Also, it can be a by‐product of different industries. For example, high levels of Se have been found in the soil near a nickel refinery in the Swansea Valley, UK (Perkins 2011). In addition, using selenium in different applications has led to released wastewater containing Se into the environment (Lemly 2004; Kavlak and Graedel 2013; Khamkhash et al. 2017; Staicu et al. 2017; Wadgaonkar, Mal, et al. 2018; Wadgaonkar, Nancharaiah, et al. 2018; Gerson et al. 2022; Lashani, Amoozegar, et al. 2023; Lashani, Moghimi, et al. 2023). For instance, the residual sludge from the electro‐refining of copper in the metallurgical industry includes 5%–25% Se (Jorgenson 2002; Wadgaonkar, Mal, et al. 2018; Wadgaonkar, Nancharaiah, et al. 2018). The four oxidation states of selenium are selenide (−2), elemental selenium (0), selenite (+4), and selenate (+6). Among them, selenate and selenite are soluble in water and, because of their toxicity and bioaccumulation potential, these species contribute to inorganic environmental contamination (Frankenberger and Engberg 1998). Symptoms of human exposure to high amounts of selenium include nail discoloration or brittleness, diarrhoea, joint pain, fatigue, hair loss, and nausea (MacFarquhar et al. 2010).

Different types of potentially toxic metals (Pb, Cu, As, Cd, Fe, and Zn) and toxic concentrations of sulfate, and nitrate at can be present and associated with selenium in these industrial wastewaters (Mal et al. 2016; Mal et al. 2021; Lashani, Amoozegar, et al. 2023; Lashani, Moghimi, et al. 2023). Thus, finding a suitable method for remediation of these pollutants is becoming increasingly imperative. For instance, a variety of potentially toxic metals, such as Hg, Pb, Cd, Cr, Cu Co, Zn, and Ni, are present in conventional fuel coal in concentrations ranging from 0.1 to 18 mg kg^−1^ (Zhang et al. 2017; Sun et al. 2019). Pb and Cd are two of these toxic metals that are listed in the top 20 hazardous substances by the United States Environmental Protection Agency (2025) and the Agency for Toxic Substances and Disease Registry (ATSDR 2012). Pb has a 150–5000 year soil persistence period, and it has been documented that Pb remains in soil more than 150 years after sludge application. Likewise, the biological half‐life of Cd is greater than 18 years (Ma et al. 2014). Numerous problems in humans, including cancer, chronic anaemia, cardiovascular diseases, cognitive impairment, kidney, bone, skin, and brain damage have been linked to toxic metal(loid) exposure (Tellez‐Plaza et al. 2013; Fu and Xi 2020; Zhang et al. 2020).

Zn is an essential trace element, but at high levels, it can be hazardous due to competition with other essential metals and disruption of metabolic processes (Plum et al. 2010). Studying the effect of these specific metals on selenite bioreduction provides a broader understanding of the adaptability and tolerance of microbial consortia in contaminated environments.

Due to their widespread presence in industrial wastewater and their recognised deleterious impact on both human health and ecosystem, Pb, Cd, and Zn were selected to investigate their effects on selenium bioreduction by a microbial consortium. These toxic metals are frequently found in wastewater from industries such as mining, battery manufacturing, and metal plating, making them significant contaminants in many environments (Tchounwou et al. 2012; Jaishankar et al. 2014). Considering their toxicity and environmental persistence, microbial communities, which play an essential role in bioremediation processes, are seriously at risk. Through investigating the interaction between these toxic metals and selenium bioreduction, we can gain substantial information about how these pollutants affect microbial activity and the effectiveness of bioremediation processes. Furthermore, Pb, Cd, and Zn are excellent choices for comprehending the various impacts of toxic metal cations on microbial consortia due to their unique chemical characteristics and toxicity processes (Gadd 2004).

Recently, there has been an increased attention on biological Se treatment approaches due to the limitations of physical and chemical procedures. In biological treatment, which is both economical and environmentally friendly, wastewater is treated using bacteria, archaea, fungi, or algae to remove selenium (Srivastava et al. 2014; Espinosa‐Ortiz et al. 2017; Wadgaonkar, Mal, et al. 2018; Wadgaonkar, Nancharaiah, et al. 2018; Wadgaonkar et al. 2019; Hosseini et al. 2023; Lashani, Amoozegar, et al. 2023; Lashani, Moghimi, et al. 2023). The presence of toxic co‐pollutants can negatively affect the microorganisms' growth and their biological activities, which lead to adverse impact on removal efficiency. Hence, using microorganisms or consortia that can tolerate different pollutants and remove selenite in this condition is worth exploring. Furthermore, halophilic/halotolerant microorganisms possess distinct physiological adaptations that make them valuable for biotechnological and industrial applications. They exhibit rapid growth rate and thrive in environments with high ion and salt concentrations, requiring minimal nutritional needs. Traditional bioremediation methods are often ineffective in saline environments due to the inhibitory effects of NaCl on these processes. Halophilic microorganisms are highly suitable for the bioremediation of toxic metals in saline and hypersaline environments because they possess specialised adaptive mechanisms that allow them to maintain cellular stability and metabolic activity under extreme osmotic stress. Their salt‐tolerant physiological systems, including efficient osmotic balance strategies and salt‐stable enzymes, enable them to function where non‐halophilic microbes fail. In addition, halophiles exhibit diverse metal‐resistance mechanisms, such as regulated transport, sequestration by biopolymers, and enzymatic detoxification, which support effective metal removal in highly saline conditions. Owing to their ability to remain active and resilient in environments with high concentrations of salts and pollutants, halophilic microorganisms represent promising candidates for sustainable bioremediation of saline wastewaters and metal‐contaminated hypersaline habitats (Amoozegar et al. 2008; Kabiri et al. 2009; Amoozegar and Siroosi 2015; Martínez‐Espinosa 2024; Rezaei et al. 2025).

Co‐pollutants have received little attention in microbial selenium‐reduction studies, likely because the simultaneous action of multiple contaminants introduces substantial complexity to interpreting microbial responses and poses significant challenges for elucidating the underlying mechanistic pathways. In this study, we used a consortium containing halophilic/halotolerant bacteria and/or yeast for simultaneous removal of selenite and toxic metals including Pb, Zn, and Cd. Also, we investigated the kinetics of selenite removal and the effect of molybdate on its reduction using microbial consortia since molybdate is the cofactor of Complex Iron–Sulfur Molybdoenzymes (CISM) (Rothery et al. 2008) which play a significant role in the reduction of selenite by these microbial consortia.

## 2 Materials and Methods

### 2.1 Microbial Sources

*Halomonas titanicae* LW94, *Bacillus toyonensis* 91, and *Cutaneotrichosporon cutaneum* 126 were isolated from saline soil samples and identified in our previous study (Lashani, Amoozegar, et al. 2023; Lashani, Moghimi, et al. 2023). *Trichosporon cutaneum* 121 and *Blastobotrys persicus* IBRC‐M 30238 129 had been isolated and reported previously (Ghorbannezhad et al. 2018; Nouri et al. 2018). The yeast *Yarrowia lipolytica* ATCC 18942 was obtained from the American Type Culture Collection. These details have now been included in the revised text.

### 2.2 Culture Condition of Microbial Consortium

In our previous study (Lashani, Amoozegar, et al. 2023; Lashani, Moghimi, et al. 2023), a microbial consortium composed of several halophilic/halotolerant selenite‐reducing bacteria and yeast was constructed. Given the high capability of yeasts for the biosorption of metals and metalloids, as well as the strong reductase enzyme activities in bacteria, a consortium composed of halophilic and halotolerant bacteria and yeasts was developed, as these microorganisms possess remarkable bioremediation potential and are well adapted to environments with high concentrations of pollutants and salts. The microbial consortium was made of two bacteria, including *H. titanicae* LW94, *B. toyonensis* 91, and 4 yeasts, including *C. cutaneum* 126, *T. cutaneum* , *B. persicus* IBRC‐M 30238 129, and *Y. lipolytica* ATCC 18942 and cultured in BSM medium (basal salt medium) containing (g/L, pH 7.5), NaCl, 50 g; KH_2_PO_4_, 0.1 g; CaCl_2_, 0.2 g; NH_4_Cl, 0.2 g; MgCl_2_.6H_2_O, 0.12 g; yeast extract, 0.5 g, and succinate 20 mM. The word “bacteria” and “yeast” showed a consortium made of all bacterial strains and yeast species, respectively, and the word “mix” refers to a consortium composed of all bacteria + yeast species. All experiments in this study have performed in three replicates and all microbial strains were preserved and kept at a temperature of −70°C for long‐term preservation at the Department of Microbiology (University of Tehran).

### 2.3 Selenite Bioreduction Assay

For quantifying selenite reduction by the microbial consortium, the method of (Li et al. 2014) was applied. In this experiment, samples were taken in specific time intervals and centrifuged at 6000×*g* for 20 min at 4°C for separating the biomass. A volume of 1 mL of the supernatants was added to 0.5 mL HCl (4 mM) and 1 mL ascorbic acid (1 M). By adding ascorbic acid as a reducing agent, selenite will transform to elemental selenium and red colour will appear in the solution. After 10 min, the absorption was measured at 500 nm by spectrophotometer (Rayleigh UV‐1601). Under acidic conditions, ascorbic acid reduces residual selenite to elemental selenium, which forms a red colloidal suspension with a characteristic absorbance maximum at 500 nm. For calibration curve, different Na_2_SeO_3_ concentrations ranging from 0 to 2.5 mM were used. The removal percentage (%) was calculated by using the below formula (Espinosa‐Ortiz et al. 2017).

$$\text{extent of removal}\;\left(\%\right)=\frac{\text{initial concentration}-\text{concentration}\;\mathrm{at}\;t1}{\text{initial concentration}}\times 100$$

### 2.4 Sequential Test for Selenite Removal by Consortia

Sequential tests for selenite removal by the MIX consortium (a consortium composed of all bacterial and yeast strains) were conducted in BSM medium containing 0.5 mM selenite under different conditions, including:

MIX: Equal ratios of each microorganism in the inoculum (comprising two bacterial and four yeast strains) based on OD_600_ from overnight culture of microorganisms.

BY: Bacteria inoculated first, followed by yeast after 2 days.

YB: Yeast inoculated first, followed by bacteria after 2 days.

2BY: A 2:1 ratio of bacteria to yeast in the inoculum.

B2Y: A 2:1 ratio of yeast to bacteria in the inoculum.

1B1Y: Equal ratios of bacteria and yeast were incubated at 30°C and 160 rpm for 5 days.

### 2.5 Kinetics of Selenite Reduction by Microbial Consortium

The kinetics of selenite bioreduction by microbial consortium were investigated based on the Nguyen et al. (2019) study. In our previous study, we confirmed the reduction of selenite is one of the main mechanisms for removal of this oxyanion using microbial consortia. In various microorganism, different types of reductase enzymes, including fumarate reductase, glutathione reductase, thioredoxin reductase, hydrogenase I, and nitrite reductase are involved in selenite and/or selenate reduction (Basaglia et al. 2007; Hunter 2014a; Hunter 2014b; Miralles‐Robledillo et al. 2019; Hou et al. 2025). Hence, in this experiment, selenite in the range of 0.75–10 mM was added to culture media, and the reduction rate was described by the Michaelis–Menten equation, as follows:

$$v=\frac{\text{Vmax}\;\left[S\right]}{\mathrm{Km}+\left[S\right]}$$

In this equation, *v* (μM h^−1^), [S] (μM), Vmax (μM h^−1^), and Km are the rate of selenite removal, initial concentration of selenite, maximum rate of selenite reduction, and Michaelis–Menten constant, respectively. The Vmax and Km were calculated through the linearized method using a Lineweaver–Burk plot (Nguyen et al. 2019) by using Excel (2021). Based on our previous results, the maximum amount of selenite reduction occurred after about 64 h after inoculation, and at this time, samples were taken, and the rate of selenite removal from culture media was measured at this time.

### 2.6 Evaluation of Biosorption by Energy‐Dispersive X‐Ray Spectra and Electron Microscopy

In this experiment, microbial species which were used for construction of consortium were cultured in media containing 0.5 mM selenite separately and incubated for 2 days at 30°C. The biomass was collected and washed twice with PBS and fixed with glutaraldehyde. After dehydration by different concentrations of ethanol (25%, 50%, 75%, and 100%) for preventing cell membrane damage in microorganisms, the samples were used for electron microscopy and energy‐dispersive x‐ray spectroscopy (EDS) analyses.

### 2.7 Effect of Molybdate on Selenite Bio Removal by Microbial Consortia

Effect of molybdate (10 mM) as a cofactor of multiple reductases was investigated on selenite removal by microbial consortia on BSM medium containing 0.5 mM selenite and compared to selenite containing flasks without molybdate. After inoculation, the flasks were incubated at 30°C, 160 rpm for 5 days. It is worth mentioning that no inhibitory effect of molybdate was observed on the growth of microbial consortia.

### 2.8 Effect of Potentially Toxic Metals on Selenite Bioreduction by Microbial Consortia

The effect of different potentially toxic metals on selenite bioreduction by the microbial consortium was investigated. In this experiment, the microbial consortium was cultured in the 100 mL flask containing 25 mL BSM culture medium in the presence of three potentially toxic metals: cadmium, zinc, and lead. The concentration of 150 mg/L of each potentially toxic metal was used separately and 50 mg/L together associated with 0.5 mM selenite. The cultures were incubated at 30°C and 160 rpm for 5 days. The samples were analysed by ICP‐OES (Varian‐735 Radial) after separating the biomass for evaluation of the removal extent of toxic metals by the microbial consortium in the presence of selenite.

### 2.9 Statistical Analysis

The results of these experiments were analysed statistically with SPSS (version 22). One‐way ANOVA was performed to compare differences among means, followed by Tukey's post hoc test for multiple comparisons. Level of *p* < 0.05 was defined as a significant difference.

## 3 Results and Discussion

### 3.1 Sequential Test for Selenite Reduction

In this experiment, different combinations of inoculum composed of bacteria and yeast were used for selenite removal and the results showed that all mix consortium combinations were able to remove Se from the media on the order of 70%–75% without any significant differences (*p* value < 0.05) over the duration of the experiment (Figure S1).

Our hypothesis for using a microbial consortium composed of bacteria and yeasts for selenite removal was based on the high biosorption capacity of yeasts (Kieliszek et al. 2015), which helps attenuate selenite toxicity, and the strong reductase activity of bacteria, which facilitates selenite reduction. The lack of observable differences among treatments may stem from the consortium's rapid progression to a similar active state and the functional redundancy among its microbial strains present in the consortium, factors whose effects become more distinguishable only over longer incubation periods.

The results of this experiment highlight the potential of microbial consortia for selenite removal, emphasising the complementary roles of bacteria and yeasts in this process. Our previous studies show the synergistic effect of these strains present in the consortium compared to each strain individually in selenite removal (Lashani, Amoozegar, et al. 2023; Lashani, Moghimi, et al. 2023). Bacteria, with their strong reductase activity, are efficient for the reduction of selenite to less toxic forms among other microorganisms (Eswayah et al. 2016), while yeasts contribute through their high biosorption capacity (Kieliszek et al. 2015). This can give superior performance of the MIX consortium, where microbial strains were inoculated in equal ratios, and underscores the importance of balancing microbial interactions to optimise selenite removal. This finding highlights the importance of optimising and exploring the microbial ratio bacteria: fungi in a consortium to avoid competition and enhance positive interactions.

### 3.2 Kinetics of Selenite Bioreduction Through the Michaelis–Menten Equation

**FIGURE 1:**
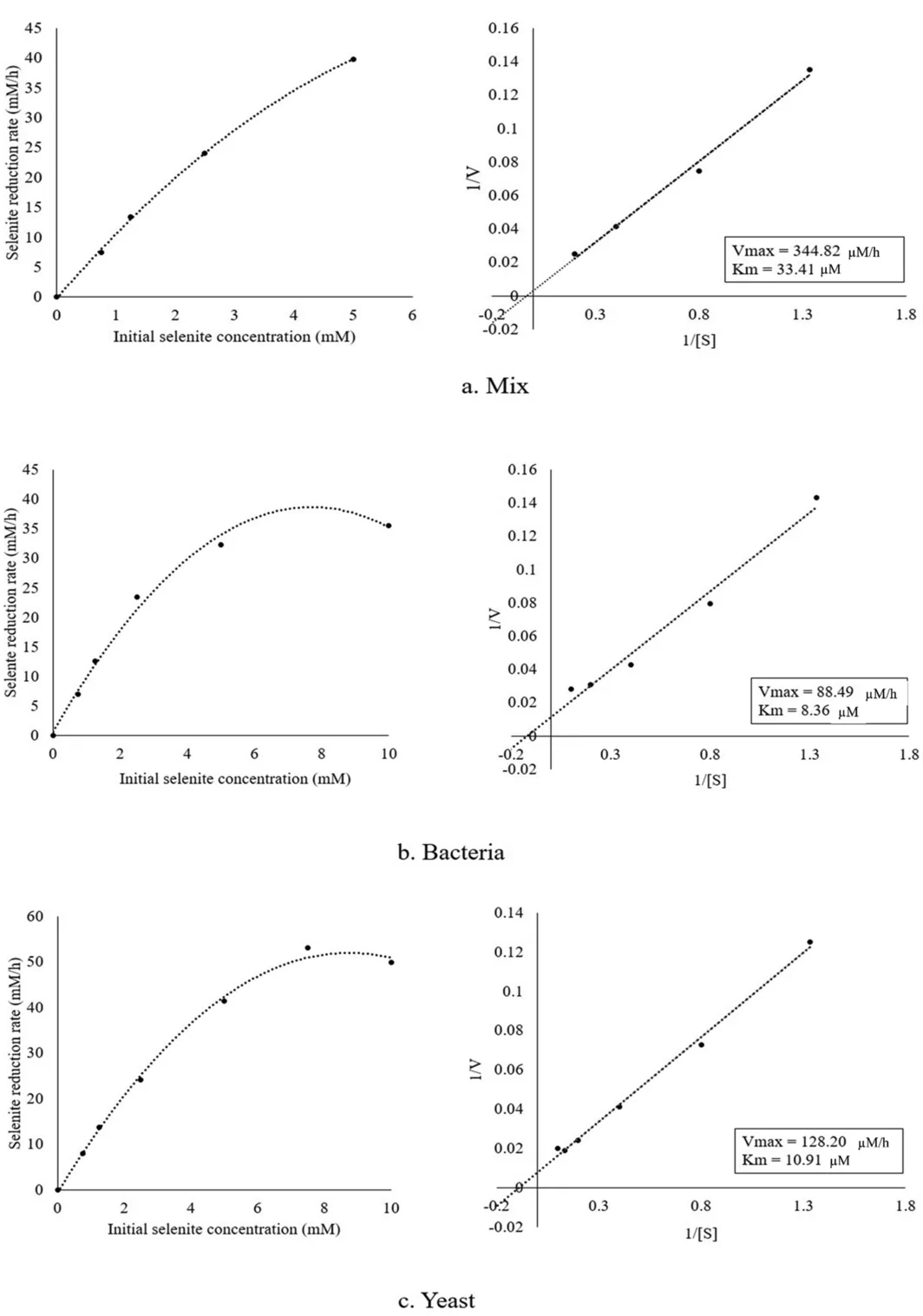
Kinetics of microbial reduction of selenite based on the Michaelis–Menten equation by three different microbial consortia, including bacteria (*R* ^2^ = 0.98, *y* = 0.0945*x* + 0.0113), yeast (*R* ^2^ = 0.9958, *y* = 0.086*x* + 0.0078), and mix (*R* ^2^ = 0.993, *y* = 0.0969*x* + 0.0029).

In this experiment, different concentrations of selenite (0.75–10 mM) were used, and a kinetic curve of the removal rate from the media was generated. The value of *R* ^2^ in the Lineweaver–Burk plots of all three consortia shows that the kinetics of selenite removal are well fitted with the Michaelis–Menten equation. The results are summarised in the Figure 1.

Microorganisms with lower Km for selenite reduce it in lower concentrations and *vice versa*. The highest rate of selenite reduction (Vmax) was observed in the order of mix culture > yeast > bacterial consortium. A lower Km of a consortium indicates that it performs better at lower selenite concentrations. Also, a higher Vmax value in the mixed consortium indicates the high ability of this consortium to remove selenite at higher concentrations. Both kinetic parameters of these three consortia, including Km and Vmax, were significantly higher than those observed in a comparative study using *Shewanella* sp. HN‐41, which was cultivated anaerobically in a culture medium containing lactate as the only carbon source and electron donor (Tam et al. 2010). The values of Vmax and Km for *Shewanella* sp. HN‐41 were reported at 1.37 mM h^−1^ and 88.0 mM respectively. Research on the kinetics of selenite reduction through the Michaelis–Menten method is very limited (Tam et al. 2010). According to Nguyen et al. (2019), *Citrobacter* sp. NVK‐2 exhibited the highest Vmax at 58.82 μM·h^−1^ with a Km of 3737.12 μM, indicating moderate substrate affinity but high reaction velocity. Conversely, *Cronobacter* sp. THL1 displayed the lowest Km at 936.82 μM, suggesting better substrate affinity but a relatively lower Vmax of 19.61 μM h^−1^. Also, Km for *Providencia* sp. NVK‐2A and *Citrobacter* sp. NVK‐6 were 3044.73 and 1300.17 μM, respectively, while their Vmax were 9.26 and 19.23 μM h^−1^ (Nguyen et al. 2019). *Shewanella* sp. HN‐41 demonstrated the highest overall Vmax at 1.37 mM h^−1^ (equivalent to 1370 μM h^−1^), though with a substantially higher Km of 88.0 mM, indicative of low substrate affinity (Tam et al. 2010). These findings reflect the diverse kinetic capabilities and substrate preferences across bacterial systems.

### 3.3 Biosorption Assay and Electron Microscopy

In this experiment, the microbial strains cultured in the presence of selenite were examined using a scanning electron microscope. It is common that microorganisms will reduce the oxyanions of selenium to elemental form resulting in nanoparticles (NPs). The purpose of SEM analysis was to test whether SeNPs were formed extracellularly. The absence of detectable SeNPs on cell surfaces in SEM images complements our previous TEM study, which demonstrated intracellular SeNP localization in these strains (Lashani, Amoozegar, et al. 2023; Lashani, Moghimi, et al. 2023). Together, these findings indicate that selenite reduction and nanoparticle nucleation occurred primarily inside cells rather than at their surface (Figure S2). Together, these findings indicate that in the studied consortium, selenite reduction and nanoparticle nucleation occur primarily within cells rather than at the cell surface.

**FIGURE 2:**
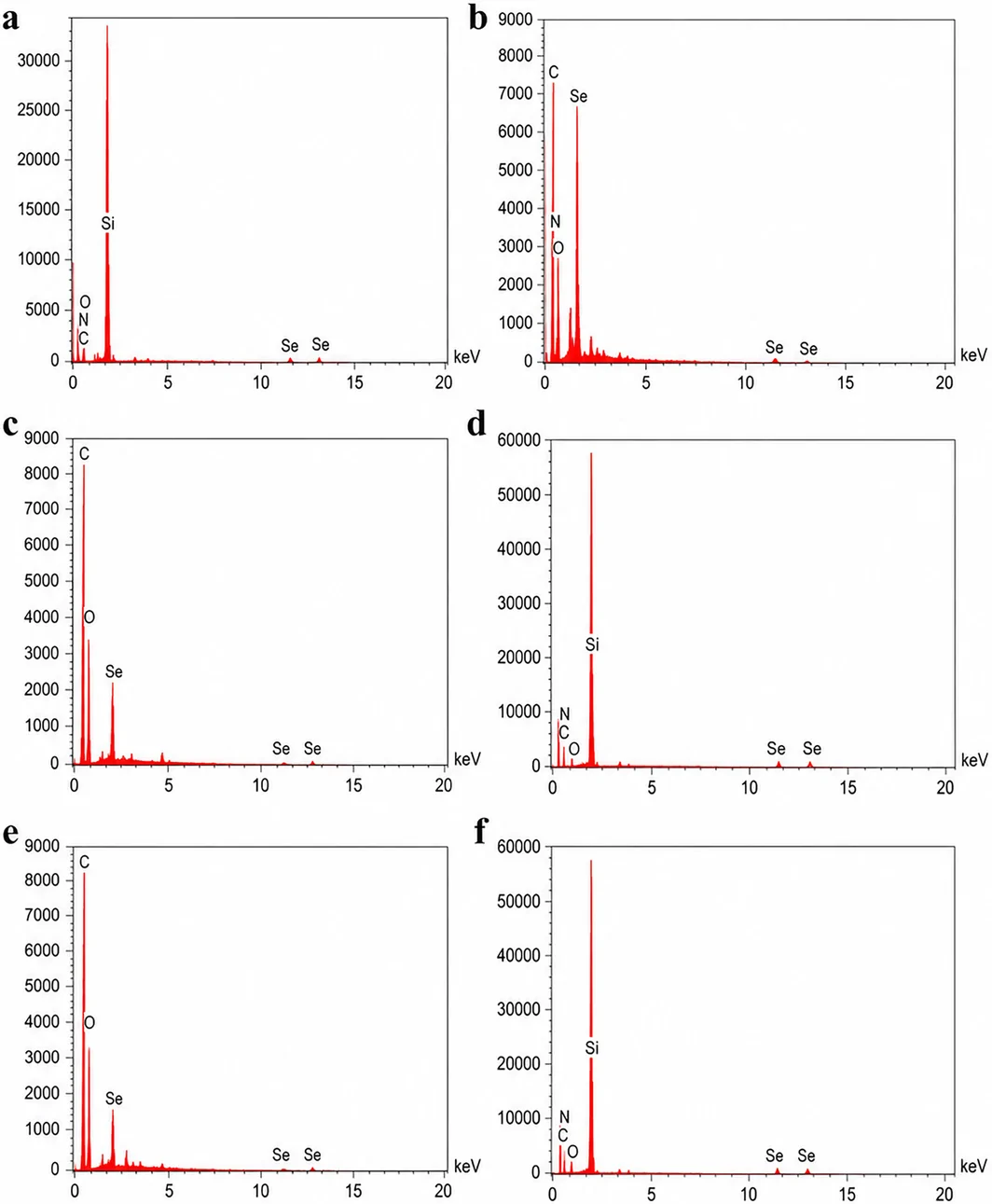
Establishing that there is Se associated with the strains present in the microbial consortium (from a–f, respectively, shows the presence of selenium in strains *Bacillus toyonensis* 91, *Halomonas titanicae* LW94, *T. cutaneum* , *Y. lipolytica*, *Blastobotrys persicus* IBRC‐M 30238 129 and *Cutaneotrichosporon cutaneum* 126) determined by EDS.

To investigate if selenite is associated intracellularly, EDS analysis was performed in order to investigate the sequestration/biosorption of selenite to the microbial strains present in the consortium. The results are shown in Figure 2, where high Se content is associated with all the cells, yet at lesser amounts in strains *T. cutaneum* and 129. This non nanoparticle Se may be in the form of RS‐Se or SeR_n_ products. It seems that bioreduction (intracellular transformation) and biosorption (surface association) likely contribute to the overall selenium removal by these microorganisms.

### 3.4 Effect of Molybdate on Selenite Bioreduction

Our previous data demonstrated an inhibitory effect of tungstate, an inhibitor of Complex Iron–Sulfur Molybdoenzymes (CISM), on selenite removal across all consortia (Lashani, Amoozegar, et al. 2023; Lashani, Moghimi, et al. 2023). Tungstate competes with molybdenum for binding to the molybdopterin cofactor in several enzymes (Pierru et al. 2006). This finding indicates that at least one mechanism involved in selenite removal by the microbial consortia utilizes this enzyme complex.

**FIGURE 3:**
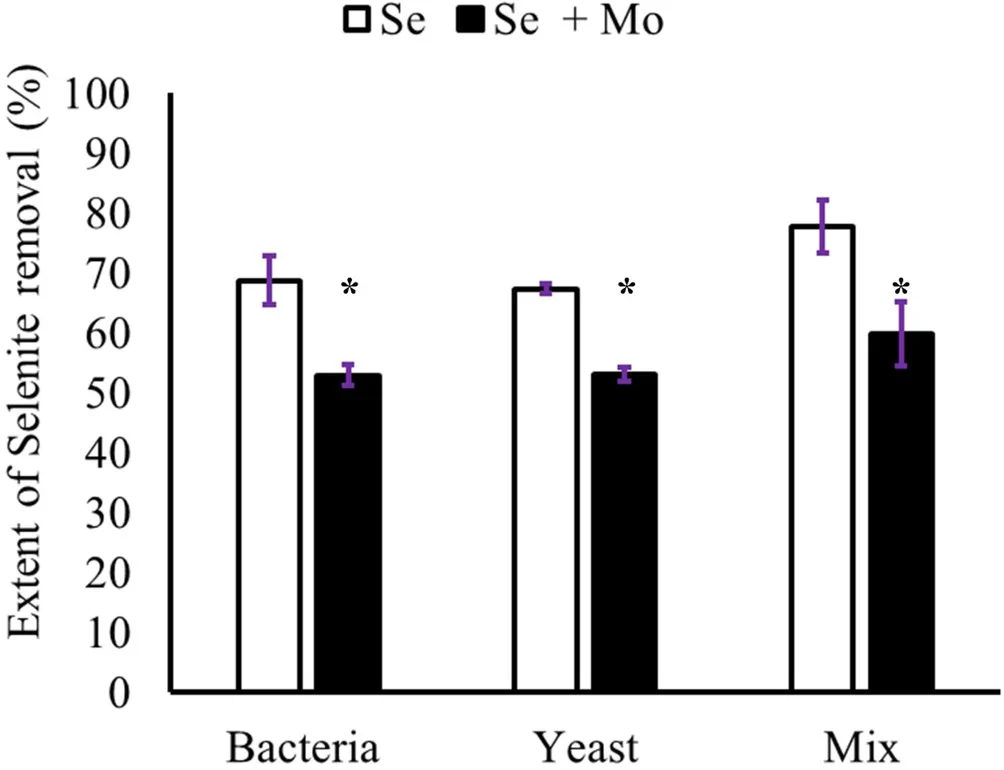
Effect of molybdate (Mo$O_{4}^{2-}$, 10 mM) on removal of selenite (0.5 mM) by various microbial consortia (Se, Se$O_{3}^{2-}$; Mo, Mo$O_{4}^{2-}$) at 30°C (*N* = 3, data were analysed by one‐way ANOVA for each consortium followed by Tukey's post hoc test, *p < 0.05).

In this study, extra molybdate (equal to the concentration of tungstate) was added to the BSM medium to investigate its effect on selenite removal by different consortia as to follow this hypothesis, more Mo available could lead to increased selenium removal. However, our results (Figure 3) showed that the ability of all consortia to remove selenite was significantly reduced in the presence of 10 mM molybdate.

Studies on the role of molybdate in microbial selenite removal are limited, but existing literature indicates that molybdate interferes with sulfate utilisation in sulfate‐reducing bacteria. Although our consortium is not sulfate‐reducing, our previous studies have shown that 20%–40% of sulfate is removed in the presence of 2–4 g/L sulfate and 0.5 mM selenite in the culture medium (Lashani, Amoozegar, et al. 2023; Lashani, Moghimi, et al. 2023). It is known that molybdate utilises sulfate transporters to enter the cell (similar to selenite) and interferes with the production of adenosine phosphosulfate, thereby depriving cells of reduced sulfur compounds (Patidar and Tare 2005), which are known to be involved in selenite reduction via the Painter reaction often through glutathione (Harrison et al. 2007; Lampis et al. 2017). Thus, although CISMs are still present, the lack of sulfur could limit the requisitions via R‐SH chemistries.

### 3.5 Effect of Metal Cations on Selenite Bioreduction

**FIGURE 4:**
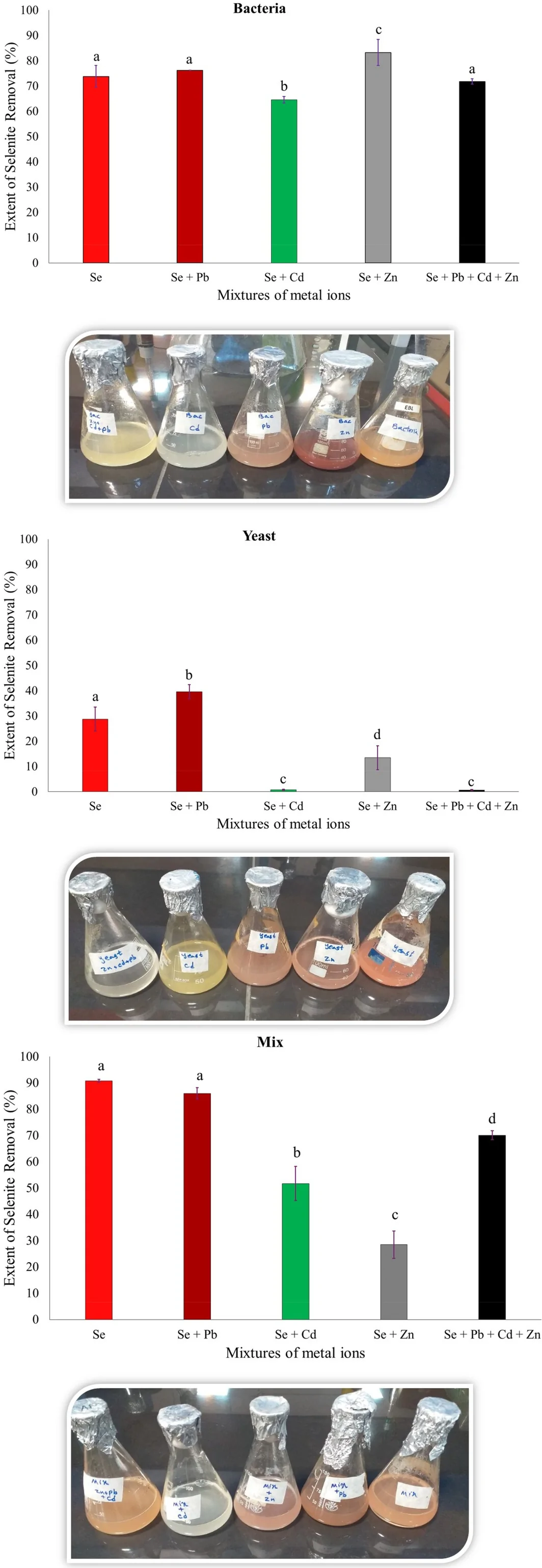
The effect of toxic metals, including lead (Pb), cadmium (Cd), and zinc (Zn) on the removal of selenite (0.5 mM = 86.47 mg/L) by three bacterial, yeast and mix consortia in the BSM culture media over 120 h (a concentration of 150, and 50 mg/L of each metals were used for separate and the combination of Zn, Cd, and Pb study, respectively). Photographs of the culture flasks provide a visual of selenite reduction reflected in the pink orange coloration (*N* = 3, different letter showed significant differences based on post hoc test).

In selenite‐contaminated industrial wastewater, there are usually various co‐polluting metals such as zinc, cadmium, and lead. There are few studies exploring whether the presence of these metal cations would affect the performance of the consortium in the removal of selenite. Therefore, in this study, the effect of these toxic metals on the removal of selenite by the microbial consortium has been investigated. In the single metal test, 150 mg/L of each cation was used (the highest concentration present in industrial wastewater), while 50 mg/L of each cation was added to culture media for the combination of all cations (Pb + Cd + Zn). The effect of metal cation co‐pollutants on the bacterial consortium had little effect on selenite removal (Figure 4). On the other hand, zinc increased the rate of selenite removal by the bacterial consortium compared to selenite on its own. In the yeast consortium, unlike cadmium and zinc, lead does not have an inhibitory effect on selenite removal. In the mixed consortium, the amount of selenite removal is the lowest in the presence of zinc, and the presence of cadmium in the culture medium reduces the amount of selenite removal. In the presence of lead, selenite removal is not significantly different from control conditions.

Sinharoy et al. (2022) investigated the effect of toxic metals such as zinc, cadmium, and copper on selenite removal in inverse fluidized bed bioreactor (IFBR). Their findings showed that the removal extent of selenite (the initial concentration of 0.45 mM–80 mg/L) decreased from 93.9% to 45.8%–85% in the presence of 50–10 mg/L of toxic metals (Sinharoy et al. 2022). In another research performed by Mal et al. (2016), the selenite removal was investigated in the presence of toxic metals, including zinc and lead using anaerobic granular sludge. In this investigation, the presence of 150–400 mg/L of zinc and lead had little effects on selenite removal (92% removal extent of selenite), while only 48%–65% of selenite was reduced in the presence of equal concentration of cadmium (Mal et al. 2016).

Zhang et al. (2022) used a complex bacterial community immobilised by alginate for the simultaneous removal of cadmium and selenite from wastewater. Their findings showed that in the presence of selenite, the removal of Cd^2+^ enhanced compared to the condition without exposure of selenite due to adsorption of Cd^2+^ to SeNPs (Zhang et al. 2022). In another study, the co‐reduction of lead cations and selenium oxyanions was investigated by using granular sludge. They suggested that in addition to Pb^2+^ sorption to SeNPs, production of HSe^−^ and its interaction with Pb^2+^ took place for simultaneous removal of both. Also, the Pb^2+^ treatment increased the selenate removal efficiency for selenium from 85% to 90% (14‐day incubation time) but did not affect selenite removal (Mal et al. 2021). An investigation of the Zn^2+^ ion adsorption on BioSeNPs (biogenic selenium nanoparticles) was carried out by Jain et al. (2015). In this study, BioSeNPs were produced using anaerobic granular sludge in an upflow anaerobic sludge blanket (UASB) reactor. Spherical amorphous nanoparticles with 80–260 nm in size and cap layer made of extracellular polymeric substances were produced after culturing the granular sludge in the medium containing lactate (2.24 g/L) as a carbon and electron sources and sodium selenite (0.86 g/L) and incubated at 30°C and pH 7.3 for 14 days. At pH values close to neutral, the Zn^2+^ ion adsorption on purified BioSeNPs occurs in two steps; at pH values close to acidic, ligand‐like (type II) processes take place. In this process, ligands facilitate cation attachment by displacing surface OH groups, minimising electrostatic repulsion, and expanding the number of active adsorption sites while preserving surface charge balance. Additionally, they suggested that the Zn^2+^ ions primarily adsorb to BioSeNPs via inner‐sphere complexation. The material's quick kinetics and more than 75% of the maximal adsorption capacity are two of the main benefits of employing BioSeNPs as an adsorbent. This makes it possible to recover the metal‐loaded BioSeNPs through simple gravity settling (Jain et al. 2015).

Cadmium exerts two types of effects: (i) binding to sulfhydryl groups in proteins and metabolites such as glutathione, which are required for thiol‐dependent reduction of selenite, and (ii) interfering with metalloenzymes by displacing or binding in place of essential metal cofactors such as Fe or Zn at their catalytic sites. Both processes lead to inhibition of the reduction of selenite (Xia et al. 2021).

## 4 Conclusion

This study elucidates the interactions between potentially toxic metals (Pb, Cd, and Zn) and the bioreduction of selenite by a microbial consortium constructed of halophilic and halotolerant bacteria and yeast. These findings indicate that the presence of toxic metals significantly impacts the efficiency of selenite reduction, with zinc enhancing and cadmium and lead inhibiting the process in a mixed microbial consortium. These results illustrate the challenges of bioremediation in multipollutant environments and the need for specialised methods that take into account the special interactions between pollutants and their differential effects on the biochemistry of the cells. Developing a successful solution for bioremediation requires an understanding of the mechanisms behind microbial removal of toxic metals, which can be obtained from kinetic data and microscopic analysis. In order to improve the effectiveness of bioreduction processes in a variety of contaminated environments, future research should concentrate on adjusting the compositions of microbial consortia and operational conditions.

## Author Contributions

**Elham Lashani:** writing – original draft, conceptualization, methodology, investigation, visualization, writing – review and editing, formal analysis, software, data curation. **Raymond J. Turner:** conceptualization, funding acquisition, methodology, writing – review and editing, project administration, supervision, resources, validation. **Hamid Moghimi:** conceptualization, funding acquisition, methodology, validation, writing – review and editing, project administration, supervision, resources. **Mohammad Ali Amoozegar:** conceptualization, methodology, supervision, validation, funding acquisition, resources, project administration, and writing – review and editing.

## Conflicts of Interest

The authors declare no conflicts of interest.

## Supporting information


**Figure S1:** Sequential tests for selenite removal by mix consortia (MIX, equal ratio of each microorganism in inoculum, including 2 bacteria and 4 yeasts, BY, inoculation of bacteria first and then yeast to the culture medium; YB, inoculation of yeast first and then bacteria; 2BY, the ratio of bacteria to yeast in the inoculum size is 2 to 1; B2Y, the ratio of yeast to bacteria in the inoculum size is 2 to 1; 1B1Y, the equal ratio of bacteria and yeast).

**Figure S2:** Scanning Electron Micrograph of bacterial, yeast and mix consortia in presence of 0.5 mM of selenite.

## Data Availability

The data that support the findings of this study are available on request from the corresponding author. The data are not publicly available due to privacy or ethical restrictions.
