# Supplementary material for: Effect of Metal Cations on Selenite Bioreduction by a Consortium of Halophilic/Halotolerant Bacteria and/or Yeast
Source: Environ Microbiol Rep. 2026 May 19;18(3):e70320. doi: 10.1111/1758-2229.70320 (PMC13580820; doi:10.1111/1758-2229.70320)
Supplement: Supplementary file 1 [file EMI4-18-e70320-s001.docx]

Supplementary Figure 1 sequential tests for selenite removal by mix consortia (MIX, equal ratio of each microorganism in inoculum, including 2 bacteria and 4 yeasts, BY, inoculation of bacteria first and then yeast to the culture medium; YB, inoculation of yeast first and then bacteria; 2BY, the ratio of bacteria to yeast in the inoculum size is 2 to 1; B2Y, the ratio of yeast to bacteria in the inoculum size is 2 to 1; 1B1Y, the equal ratio of bacteria and yeast)
