# Supplementary material for: Effect of Metal Cations on Selenite Bioreduction by a Consortium of Halophilic/Halotolerant Bacteria and/or Yeast
Source: Environ Microbiol Rep. 2026 May 19;18(3):e70320. doi: 10.1111/1758-2229.70320 (PMC13580820; doi:10.1111/1758-2229.70320)
Supplement: Supplementary file 2 [file EMI4-18-e70320-s002.docx]

Supplementary Figure 2. Scanning Electron Micrograph of bacterial, yeast and mix consortia in presence of 0.5 mM of selenite
